# Differential long-term bivalent HPV vaccine cross-protection by variants in the Costa Rica HPV vaccine trial

**DOI:** 10.1038/s41541-024-00896-y

**Published:** 2024-06-08

**Authors:** Jaimie Z. Shing, Carolina Porras, Maísa Pinheiro, Rolando Herrero, Allan Hildesheim, Danping Liu, Mitchell H. Gail, Byron Romero, John T. Schiller, Michael Zúñiga, Sambit Mishra, Laurie Burdette, Kristine Jones, John Schussler, Rebeca Ocampo, Jianwen Fang, Zhiwei Liu, Douglas R. Lowy, Sabrina H. Tsang, Ana Cecilia Rodríguez, Mark Schiffman, Cameron B. Haas, Loretto J. Carvajal, Jalen R. Brown, Aimée R. Kreimer, Lisa Mirabello, Costa Rica HPV Vaccine Trial (CVT) Group, Bernal Cortés, Paula González, Rolando Herrero, Silvia E. Jiménez, Carolina Porras, Ana Cecilia Rodríguez, Allan Hildesheim, Aimée R. Kreimer, Douglas R. Lowy, Mark Schiffman, John T. Schiller, Mark Sherman, Sholom Wacholder, Ligia A. Pinto, Troy J. Kemp, Mary K. Sidawy, Wim Quint, Leen-Jan van Doorn, Linda Struijk, Joel M. Palefsky, Teresa M. Darragh, Mark H. Stoler

**Affiliations:** 1 Division of Cancer Epidemiology and Genetics, National Cancer Institute, Rockville, MD USA; 2 Agencia Costarricense de Investigaciones Biomédicas (ACIB), formerly Proyecto Epidemiológico Guanacaste, Fundación INCIENSA, San José, Costa Rica; 3 Center for Cancer Research, National Cancer Institute, Bethesda, MD USA; 4 Leidos Biomedical Research, Inc, Frederick National Laboratory for Cancer Research, Frederick, MD USA; 5 Information Management Services Inc, Silver Spring, MD USA; 6 Division of Cancer Treatment and Diagnosis, National Cancer Institute, Bethesda, MD USA; 7 Independent consultant, San José, Costa Rica; 8 Georgetown University, Washington, DC USA; 9 DDL Diagnostic Laboratory, Rijswijk, Netherlands; 10 University of California, San Francisco, CA USA; 11 University of Virginia, Charlottesville, VA USA

**Keywords:** Vaccines, Infectious diseases

## Abstract

The AS04-adjuvanted human papillomavirus (HPV)16/18 vaccine, an L1-based vaccine, provides strong vaccine efficacy (VE) against vaccine-targeted type infections, and partial cross-protection to phylogenetically-related types, which may be affected by variant-level heterogeneity. We compared VE against incident HPV31, 33, 35, and 45 detections between lineages and SNPs in the L1 region among 2846 HPV-vaccinated and 5465 HPV-unvaccinated women through 11-years of follow-up in the Costa Rica HPV Vaccine Trial. VE was lower against HPV31-lineage-B (VE=60.7%;95%CI = 23.4%,82.8%) compared to HPV31-lineage-A (VE=94.3%;95%CI = 83.7%,100.0%) (VE-ratio = 0.64;95%CI = 0.25,0.90). Differential VE was observed at several lineage-associated HPV31-L1-SNPs, including a nonsynonymous substitution at position 6372 on the FG-loop, an important neutralization domain. For HPV35, the only SNP-level difference was at position 5939 on the DE-loop, with significant VE against nucleotide-G (VE=65.0%;95%CI = 28.0,87.8) but not for more the common nucleotide-A (VE=7.4%;95%CI = −34.1,36.7). Because of the known heterogeneity in precancer/cancer risk across cross-protected HPV genotype variants by race and region, our results of differential variant-level AS04-adjuvanted HPV16/18 vaccine efficacy has global health implications.

## Introduction

The prophylactic AS04-adjuvanted human papillomavirus (HPV)16/18 vaccine (Cervarix^®^, GlaxoSmithKline Biologicals; Rixensart, Belgium) contains virus-like particles of HPV16/18 that self-assemble from pentamers of the L1 major capsid proteins, displaying epitopes that induce high titers of neutralizing antibodies^1^. These antibodies provide strong protection against vaccine-type infections in HPV-naïve people^2,3^. Some carcinogenic HPV genotypes have similarities in epitopes to vaccine-targeted genotypes, which allow for partially cross-reactive antibody responses^4,5^. Namely, HPV31, 33, and 35 are phylogenetically related to HPV16, while HPV45 is phylogenetically related to HPV18^6,7^. Due to these similarities, Cervarix provides moderate cross-protection against HPV31, 33, 45, and possibly 35^8,9^. Results from the Costa Rica HPV Vaccine Trial (CVT) demonstrate a significant combined vaccine efficacy (VE) of 64% against HPV31/33/45 two to eleven years following three doses of Cervarix and a significant VE of 23% against HPV35 among women without evidence of prevalent infection by these types at the time of vaccination^8^.

However, VE against cross-protected types may be influenced by viral genetic variation in lineages, sublineages, and/or single nucleotide polymorphisms (SNPs) within each type, all of which are referred to as “variants.” HPV lineages are distinct groups of evolutionarily related viral isolates within an HPV type, usually visualized as a branch on a phylogenetic tree, that differ from one another by 1%-10% of their L1 genome sequence^7,10^. Nested within lineages of a given type are evolutionarily related sublineages that differ from one another by 0.5%–1% of the whole genome^7,10^. Lineages and sublineages are defined by a unique set of stable, highly correlated nucleotide changes across the viral genome (termed lineage-associated SNPs). HPV variation can also occur as individual SNPs (variation not linked to a lineage or sublineage), and recent HPV genome sequencing studies have revealed that high-risk HPV types have tremendous genome diversity^11–15^. HPV genetic variation at each of these levels includes genetic changes in the relatively conserved L1 gene region, potentially leading to heterogeneity of the efficacy of Cervarix, an L1-based vaccine. For instance, some lineages of HPV31 and 45 have varying degrees of sensitivity to neutralization by vaccine-induced antibodies^4,16^, which may be attributed to one L1 residue changing the structural conformation of the capsid protein, increasing vaccine-induced antibody recognition^4^.

Epidemiological data from vaccine trials are invaluable to evaluate whether variant-level differences in VE may explain partial protection for specific types. We previously evaluated differences in the cross-protection of Cervarix by lineage during four years following vaccination^17^. Here, we expand our prior analysis by evaluating VE of Cervarix across lineages and L1-SNPs of HPV31, 33, 35, and 45 through 11 years post-vaccination in CVT.

## Results

**Fig. 1:**
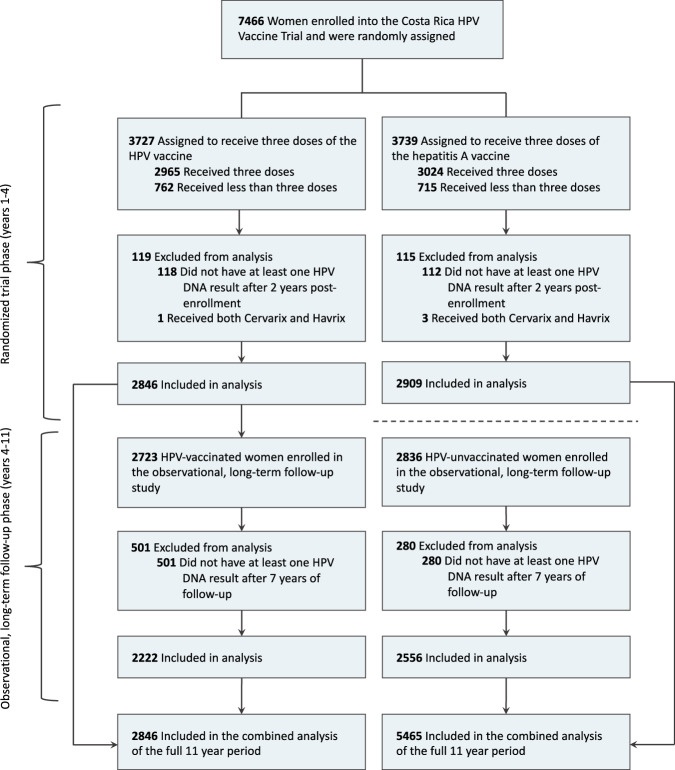
Flow diagram of included study participants in the analytical cohort. This figure describes the flow of study participants included in the analysis from both the randomized trial phase of the Costa Rica HPV Vaccine Trial and the observational long-term follow-up phase through 11 years of follow up. The final analytical sample for the HPV vaccinated group and HPV-unvaccinated group for the full 11-year period is shown in the bottom most boxes.

**Fig. 2:**
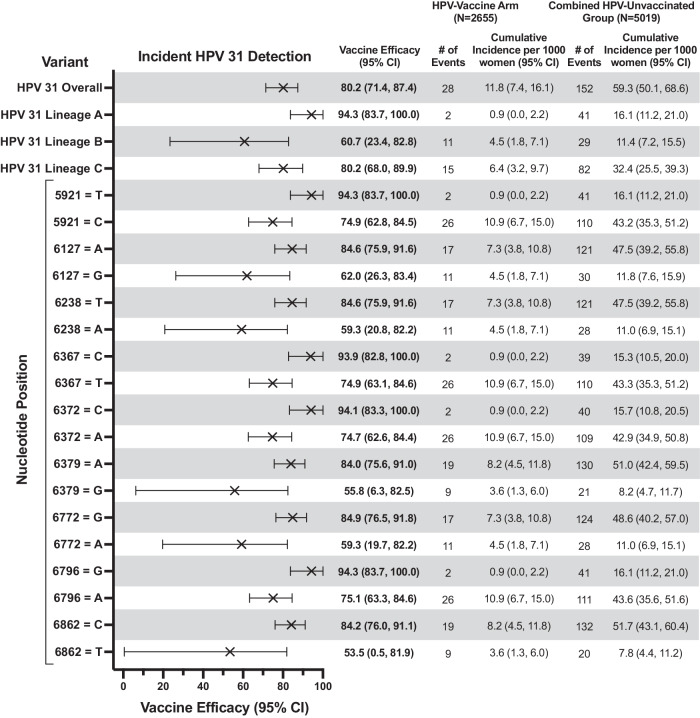
HPV vaccine efficacy against HPV31 variants through 11 years post-vaccination. This figure shows the HPV vaccine efficacy, indicated by the “X” symbol, against incident HPV31 detections overall and by lineage and SNP, and the corresponding 95% CIs. The bolded estimates indicate statistical significance. For each SNP, both nucleotides are shown. The analytical cohort includes all women who received all three doses of either Cervarix or Havrix, and all women in the unvaccinated control group who had at least one HPV DNA test result after two years post-enrollment and who did not have an outcome of interest during the initial two-year period. CI confidence interval, HPV human papillomavirus, SNP single nucleotide polymorphism.

**Fig. 3:**
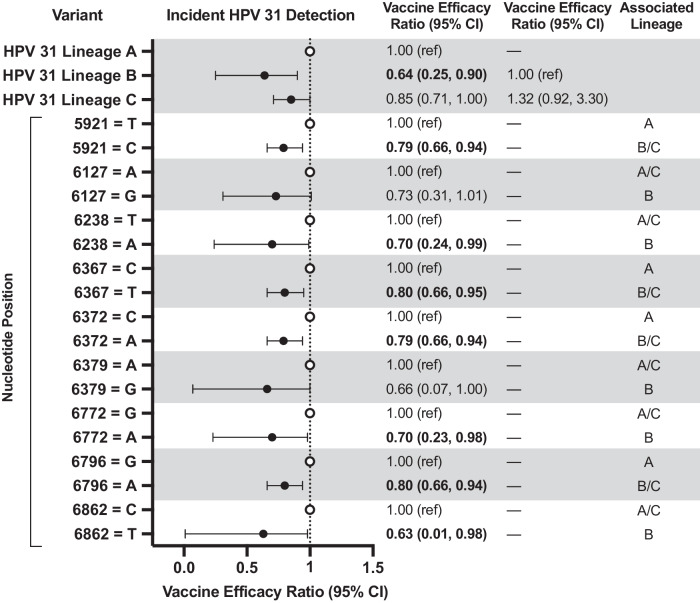
HPV vaccine efficacy ratio comparing efficacy between HPV31 lineages and L1 SNPs through 11 years post-vaccination in the Costa Rica HPV Vaccine Trial. This figure shows the HPV vaccine efficacy ratios, indicated by the circle symbols, against incident HPV31 detections by lineage and SNP, and the corresponding 95% CIs. The bolded estimates indicate statistical significance. For each SNP, both nucleotides are shown. The closed circles represent the vaccine efficacy ratio comparing vaccine efficacy against a specific variant compared to the vaccine efficacy against the reference group (open circles). The analytical cohort includes all women who received all three doses of either Cervarix or Havrix, and all women in the unvaccinated control group, who had at least one HPV DNA test result after two years post-enrollment and who did not have an outcome of interest during the initial two-year period. CI confidence interval, HPV human papillomavirus; SNP single nucleotide polymorphism.

The analytical cohort included 2846 HPV-vaccinated and 5465 HPV-unvaccinated women [2909 from the HAV-vaccine arm and 2556 from the unvaccinated control group (UCG)] (Fig. 1). At the fourth-year study visit, both groups had the same median age (26 years, interquartile range = 24–28 years) (Table 1). Overall, the cumulative incidence of HPV31 detection in HPV-vaccinated women was 11.8 (95%CI = 7.4,16.1) per 1000 women compared to 59.3 (95%CI = 50.1,68.6) in HPV-unvaccinated women, corresponding to a VE of 80.2% (95%CI = 71.4%,87.4%) (Fig. 2). By lineage, vaccine protection against HPV31-lineage-B (VE = 60.7%;95%CI = 23.4%,82.8%) was lower compared to HPV31-lineage-A (VE = 94.3%;95%CI = 83.7%,100.0%) (VE ratio = 0.64;95%CI = 0.25,0.90) (Figs. 2, 3). VE against HPV31-lineage C-was 80.2% (95%CI = 68.0%,89.9%) and did not differ from lineage-A (VE ratio = 0.85; 95%CI = 0.71,1.00) or lineage-B (VE ratio = 1.32;95%CI = 0.92,3.30). VE against HPV31 detection differed for several L1-SNPs, all of which were lineage-associated (i.e., part of the known linked haplotype that defines one or more of the lineages), including positions 5921, 6238, 6367, 6372, 6772, 6796, and 6862 (Fig. 3). At all of these positions, VE was higher for nucleotides that were associated with lineages-A or A/C compared to lineage-B, or lineage-A alone compared to lineages-B/C.

**Table 1:** Characteristics of women at year 4 (visit 48) by vaccination status

		HPV-Unvaccinated		
Characteristic	HPV-Vaccine Arm	Combined HPV-Unvaccinated Group	HAV-Vaccine Arm	UCG
Total, *N*	2846	5465	2909	2556
Age, years, *n* (%)				
< 26	1333 (46.8)	2457 (45.0)	1344 (46.2)	1113 (43.5)
26+	1390 (48.8)	2883 (52.8)	1440 (49.5)	1443 (56.5)
No visit	123 (4.3)	125 (2.3)	125 (4.3)	0 (0.0)
Median, IQR (range)	26, 24–28(22–32)	26, 24–28(21–32)	26, 24–28(22–32)	26, 24–28(21–32)
Marital status, *n* (%)				
Single	900 (31.6)	1469 (26.9)	884 (30.4)	585 (22.9)
Married	1650 (58.0)	3547 (64.9)	1721 (59.2)	1826 (71.4)
Widowed/divorced	164 (5.8)	302 (5.5)	163 (5.6)	139 (5.4)
No answer	132 (4.6)	147 (2.7)	141 (4.8)	6 (0.2)
Lifetime sexual partners, *n* (%)				
0	176 (6.2)	231 (4.2)	172 (5.9)	59 (2.3)
1	710 (24.9)	1487 (27.2)	742 (25.5)	745 (29.1)
2	494 (17.4)	1136 (20.8)	544 (18.7)	592 (23.2)
3+	1342 (47.2)	2470 (45.2)	1326 (45.6)	1144 (44.8)
No answer	124 (4.4)	141 (2.6)	125 (4.3)	16 (0.6)
HPV positivity, *n* (%)				
HPV16/18	57 (2.0)	418 (7.6)	197 (6.8)	221 (8.6)
Other oncogenic HPV	497 (17.5)	1092 (20.0)	574 (19.7)	518 (20.3)
Non oncogenic HPV	691 (24.3)	1199 (21.9)	662 (22.8)	537 (21.0)
No HPV	1651 (58.0)	3159 (57.8)	1625 (55.9)	1534 (60.0)
No result	125 (4.4)	131 (2.4)	129 (4.4)	2 (0.1)
Number of cross-protected type 31/33/35/45 infections for which the woman joined the analysis, *n* (%)				
1	194 (6.8)	467 (8.5)	235 (8.1)	232 (9.1)
2	23 (0.8)	66 (1.2)	40 (1.4)	26 (1.0)
3	2 (0.1)	5 (0.1)	2 (0.1)	3 (0.1)

The sum of the percentages for HPV positivity does not equal 100 because women who had multiple HPV type infections were counted in multiple rows. *HAV* Hepatitis A virus, *HPV* Human papillomavirus, *IQR* interquartile range, *UCG* screening-only, observational unvaccinated control group.

**Fig. 4:**
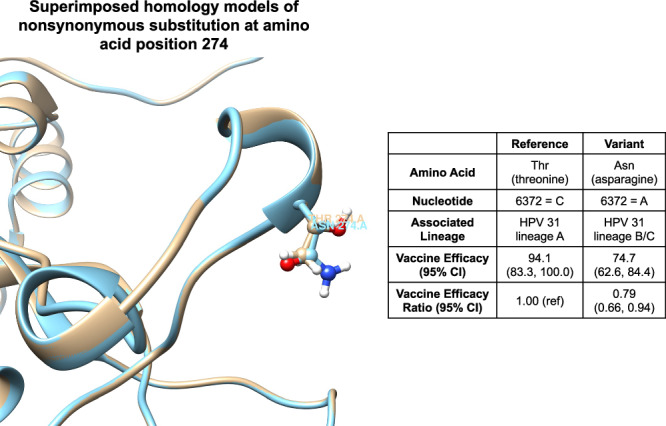
Enlarged view of superimposed homology models of HPV31 L1 illustrating the amino acid change at position 274. This figure shows the superimposed homology models of HPV31 L1, which has an amino acid change at position 274 from Thr (threonine) to Asn (asparagine), and presents the vaccine efficacy estimates for each amino acid with the vaccine efficacy ratio between the two. The tan color on the figure represents threonine, while the blue color represents asparagine. CI confidence interval, HPV human papillomavirus.

HPV31 L1-SNP position 6372 was nonsynonymous and resulted in an amino acid change from Threonine (Thr) to asparagine (Asn) at position 274 (T > N) (Table 2). Our superimposed homology models illustrate that this position is located on the FG neutralization loop (Fig. 4). Both Thr and Asn residues are polar but not charged and can form hydrogen bonds, but their side chains differ, as they belong to different amino acid functional groups (hydroxyl versus amide).

**Table 2:** Annotation of the significant HPV31 and HPV35 L1 SNPs

Nucleotide Position	Substitution	DNA Change	Codon Change	Amino Acid Position	L1 Loop	Amino Acid Change
HPV31						
5921	Synonymous	T > C	TTA > CTA	124	DE	L > L
6127	Synonymous	A > G	AAA > AAG	129		K > K
6238	Synonymous	T > A	ATT > ATA	229		I > I
6367	Synonymous	C > T	GTC > GTT	272	FG	V > V
6372	Non-Synonymous	C > A	ACT > AAT	274	FG	T > N
6379	Synonymous	A > G	TTA > TTG	276	FG	L > L
6772	Synonymous	G > A	TTG > TTA	407		L > L
6796	Synonymous	G > A	TTG > TTA	415		L > L
6862	Synonymous	C > T	CCC > CCT	437		P > P
HPV35						
5939	Synonymous	G > A	TTG > TTA	113	DE	L > L

*HPV* Human papillomavirus, *SNP* single nucleotide polymorphism.

**Fig. 5:**
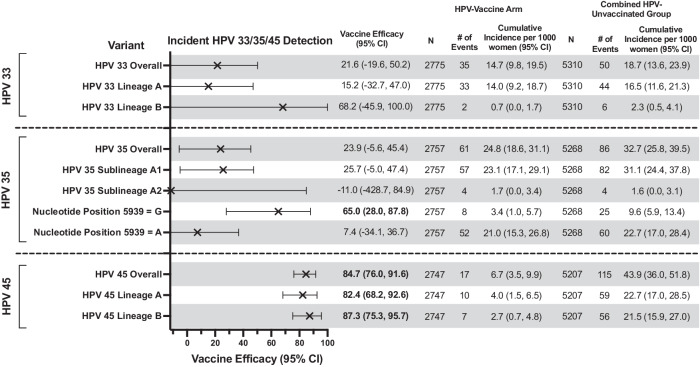
HPV vaccine efficacy against HPV33, 35, and 45 variants through 11 years post-vaccination in the Costa Rica HPV Vaccine Trial. This figure shows the HPV vaccine efficacy, indicated by the “X” symbol, against incident HPV33, 35, and 45 detections overall and by variant, and the corresponding 95% CIs. The bolded estimates indicate statistical significance. The analytical cohort includes all women who received all three doses of either Cervarix or Havrix, and all women in the unvaccinated control group, who had at least one HPV DNA test result after two years post-enrollment and who did not have an outcome of interest during the initial two-year period. CI confidence interval, HPV human papillomavirus; SNP single nucleotide polymorphism.

**Fig. 6:**
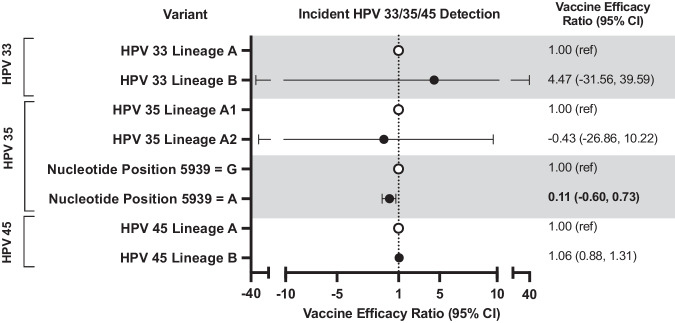
HPV vaccine efficacy ratio comparing efficacy between HPV33, 35, and 45 lineages and L1 SNPs through 11 years post-vaccination in the Costa Rica HPV Vaccine Trial. This figure shows the HPV vaccine efficacy ratios, indicated by the circle symbols, against incident HPV33, 35, and 45 detections by variant, and the corresponding 95% CIs. The bolded estimate indicates statistical significance. The closed circles represent the vaccine efficacy ratio comparing vaccine efficacy against a specific variant compared to the vaccine efficacy against the reference group (open circles). The analytical cohort includes all women who received all three doses of either Cervarix or Havrix, and all women in the unvaccinated control group, who had at least one HPV DNA test result after two years post-enrollment and who did not have an outcome of interest during the initial two-year period. CI confidence interval, HPV Human papillomavirus; SNP single nucleotide polymorphism.

The overall cumulative incidence of incident HPV33 and 35 detection was similar between women in the HPV-vaccine arm and HPV-unvaccinated group, resulting in a lack of significant VE (Fig. 5). The lineage/sublineage-stratified analyses for HPV33 and 35 were underpowered due to small case counts of HPV33-lineage-B infections and HPV35-sublineage-A2 infections, irrespective of vaccination group. While no differential VE was observed for HPV35 by sublineage, differences in VE were detected at L1-SNP position 5939 (VE-ratio = 0.11;95%CI = -0.60,0.73) (Fig. 6), a synonymous substitution at amino acid position 113 located on the DE loop (Table 2). The G nucleotide of SNP 5939 had a significant VE of 65.0% (95%CI = 28.0%,87.8%), while VE was not significant for the more common A nucleotide (VE = 7.4%;95%CI = −34.1%,36.7%) (Fig. 5). This is an independent SNP that is not associated with a sublineage. For HPV45, high VE was observed against incident detection overall (VE = 84.7%;95%CI = 76.0,91.6) (Fig. 5) and remained similarly high for lineages-A and B (Fig. 6).

## Discussion

In this analysis of long-term variant-level cross-protection of the AS04-adjuvanted bivalent HPV vaccine, we discovered that overall cross-protection against incident HPV31 detection is weighted by the very high VE against lineage-A (94%) and partial protection against lineage-B (60%) and lineage-C (80%). We identified a potentially important HPV31 SNP at position 6372, located within the FG loop of L1, which is an essential neutralization domain^18–20^. We also observed differential VE against HPV35 at SNP position 5939, which is located within another neutralization region although, this substitution was synonymous and its potential function is unclear. Lastly, we confirmed VE against HPV45 was high ( > 80%) and did not significantly differ across lineages.

The significant non-synonymous substitution we identified within the HPV31 L1 region likely resulted in changes in epitopes recognized by the vaccine-induced antibodies, leading to variant-level differences in protection; however, we did not conduct analyses of in vitro neutralization of variant-specific pseudoviruses to confirm that the amino acid change was the exact mechanism for differential VE. We observed that the Thr residue at position 274, which is associated with HPV31-lineage-A, had significantly higher VE compared to the Asn residue, which was present only in HPV31-lineages-B/C. A study noted that although the change at position 274 from Thr to Asn is relatively subtle, it still resulted in small local structural changes that could contribute to differences in distal epitope recognition because of its location on the tip of the L1 FG loop^4^. Our observation of significantly different VE between variants at this position suggests that this particular nonsynonymous substitution may partially explain heterogeneity in cross-protection against HPV31 infections by lineage.

Our discovery of variant-level differences in VE is clinically important because of heterogeneity in risk of precancer/cancer reported for cross-protected HPV genotype lineages/sublineages^11,15,21,22^. Both HPV31-lineages-A and B are associated with increased precancer/cancer risk compared to lineage-C^15^, thus, our observation of high VE against HPV31-lineage-A is promising but, lower VE against HPV31-lineage-B is concerning.

For HPV35, prior studies have shown that cervical precancer/cancer risk may differ via interactions of sublineage and race, with A1 associated with higher risk of CIN2+ among White women, and A2 being more prevalent and associated with CIN3+ among African-American women^11,23^. Our HPV35 sublineage-stratified analysis was limited by the low prevalence of one of the two sublineages in Costa Rica. However, we note at the SNP-level, VE differed at HPV35 L1-SNP position 5939, with significantly moderate VE against nucleotide-G but, a lack of significant VE against the more common nucleotide-A. While this observation would have important implications due to the possibility of HPV35-caused cervical cancer in world regions with high cervical cancer burden, this position was synonymous, meaning the biological mechanism for how it impacted VE is unclear. In a previous large characterization of HPV35 variation, we observed that nucleotide-A at HPV35 position 5939 (for which we observed no significant VE) was present in all women with an A2 sublineage infection and variably present in women with an A1 sublineage infection^11,23^, emphasizing the high prevalence of this nucleotide among people with HPV35 infections. Therefore, if differential variant-level VE against HPV35 is true, it poses important implications for global HPV vaccine implementation, particularly for Sub-Saharan African countries, where HPV35 may cause up to 10% of cervical cancers^24^, and where much of the cervical cancer burden resides^25^.

Given the long-term follow-up of our study, our results inherently demonstrate that cross-protection afforded by the AS04-adjuvanted bivalent HPV vaccine persists for at least 11 years, particularly for HPV31 and 45 infections. This conclusion supports our recent evaluation of the durability of cross-protection of Cervarix, which showed no evidence of waning cross-protection over 11 years^8^. These observations differ from prior studies that have suggested decreasing VE against HPV31 and 45 infections over time^26^.

Our study was limited by the low prevalence of HPV33-lineage-B and HPV35-sublineage-A2 in Costa Rica, resulting in a lack of power to detect differences in VE between HPV33 and 35 lineages/sublineages. We only included women with three doses because the one-dose group was limited in sample size and variant-level outcomes were not available for this group; however, one-dose cross-protection against some HPV genotypes has been observed for Cervarix, with strong protection durability over time, which provides positive global health implications^8^. Another limitation is that the SNP-level findings were predetermined in the discovery phase due to restricting to L1-SNPs with different allele frequencies by vaccination status. In this work, we took an exploratory approach and did not adjust for multiple comparisons; this investigation should be replicated in independent datasets, especially in world regions with higher prevalence of HPV33 and 35. An important consideration is that our outcomes of interests were incident, HPV31, 33, 35, and 45 detections, rather than persistence. We calculated cumulative incidence over 11-years of follow-up, which is a different metric than what is presented in global reports of HPV genotype distributions which report prevalence estimates. The majority (84%) of the Costa Rican population is White or Mestizo; thus, our results may not be generalizable to populations with more racial and ethnic diversity.

While our findings are robust and valid, VE estimates may vary depending on the prevalence of HPV genotypes and variants in the population, as the distribution of HPV genotypes and their variants differ by region, race, and ethnicity^11,15^. Specifically, the global prevalence of HPV31, 33, 35, and 45 infections ranges from 0.5%-1.2% in women with normal cytology^27^ and 1.0%–1.8% in men^28^, depending on the HPV genotype. In Costa Rica, the prevalence of HPV31, 33, 35, and 45 infections ranges from 0.5%-1.1% in women with normal cytology, which is comparable to the global prevalence^27^. Among women with normal cytology, the highest HPV prevalence is in Oceania (22%) and Africa (21%)^29^, but the distribution of HPV lineages/sublineages varies by world region^15,29^. Additionally, as noted above, HPV genotype distributions and their variants vary by race and ethnicity, such as HPV35 being more common among women with African ancestry^11,15^.

This is the most robust analysis of HPV VE by cross-protected genotype variants with the longest follow-up of HPV-vaccinated women. We used a longer exclusion period for event-counting (2 years) to reduce the potential for misclassifying prevalent infections present at enrollment. We comprehensively utilized viral whole-genome sequencing, instead of HPV targeted sequencing of the URR/E6 regions like in our prior analysis^17^, enabling precise classification of lineages/sublineages and allowing us to evaluate associations with individual nucleotide variants in the L1 sequence. We also focused solely on L1-SNPs to reduce the possibility of false positive chance findings, as the L1 region is highly relevant to analyses of the AS04-adjuvanted bivalent HPV vaccine, an L1-based vaccine. Although variations in other regions of the HPV genome, such as L2, can also affect endpoints, these variations would presumably apply to infections in both vaccinated and unvaccinated groups (i.e., non-differential). Because the HPV vaccine is composed on the L1 protein alone, variations in the L1 gene region might directly affect vaccine protection by impacting the sterilizing immunity induced by vaccination.

Our observations would not be applicable for multivalent HPV vaccines that directly target these cross-protected HPV genotypes and for higher valency HPV vaccines that use the AS04-adjuvant, which is the proposed mechanism that provides cross-protection of phylogenetically related types. Currently, a candidate adjuvanted nonavalent HPV vaccine targeting HPV6/11/16/18/31/33/45/52/58 is being evaluated^30^, which might offer even greater cross-protection than the AS04-adjuvanted bivalent HPV vaccine given the inclusion of more HPV types in the nonavalent vaccine that may offer increased opportunity for similarities with more phylogenetically related HPV types. However, many countries have used the bivalent HPV vaccine for many years and continue to use this vaccine; thus, our observations are still relevant for many birth cohorts with bivalent HPV vaccination. Importantly, the phenomenon regarding differential protection afforded by the AS04-adjuvanted bivalent HPV vaccine against variants of HPV31 and 35 is interesting and warrants further investigation, particularly in other vaccine clinical trials for external validation.

## Methods

### CVT study design

CVT is a double-blinded, randomized phase III clinical trial aimed to evaluate the efficacy of Cervarix against HPV infections and HPV-associated neoplasia^31^. During 2004–2005, before Cervarix licensure, 7466 Costa Rican women aged 18–25 years who resided in Guanacaste and Puntarenas provinces were enrolled and randomized (1:1) to receive three doses of either Cervarix or the control hepatitis A virus (HAV) Havrix^®^ vaccine, administered intramuscularly in 0.5 mL doses at 0, 1, and 6 months. Randomization was conducted using a blocked randomization method with permuted block sizes of 14, 16, and 18. After four years, participants in the HPV-vaccine arm were invited to enroll in the observational long-term follow-up phase, extending follow-up through 11 years^32^. Women in the HAV-vaccine arm were offered the HPV vaccine before exiting the study and a new screening-only cohort was recruited and enrolled, including HPV-unvaccinated women from similar birth cohorts and geographical regions as women in the original control arm. The new control group is referred to as the “unvaccinated control group” (UCG). These studies are registered with ClinicalTrials.gov, NCT00128661 and NCT00867464. All participants provided written, informed consent. All research activity for CVT was approved by Institutional Review Boards of Instituto Costarricense de Investigación y Enseñanza en Nutrición y Salud in Costa Rica and the US National Cancer Institute (Bethesda, MD, USA).

### Sample collection

During the randomized trial phase, serum samples were collected at enrollment and at annual follow-up visits. For sexually experienced women, cervical samples were collected using a Cervex-Brush (Rovers Medical Devices BV, Oss, Netherlands) and rinsed in PreservCyt solution (Hologic, Marlborough, MA, USA) for cytology and HPV DNA testing. During the observational long-term follow-up phase (LTFU), participants in both the HPV-vaccine arm and unvaccinated control group (UCG) were seen biennially, and cervical samples were collected at each of these routine clinic visits. In both the trial phase and the LTFU phase, women with low-grade cervical abnormalities were seen every six months, while women with evidence of high-grade cervical abnormalities were referred to colposcopy for evaluation and treatment, as needed.

### Outcomes

The primary outcomes were incident detection of HPV31, 33, 35, and/or 45 in cervical exfoliated cells. Each HPV-type was treated as an independent analysis; women who were infected with multiple cross-protected HPV types could contribute to each analysis. Lineage and SNPs were only determined at a single point (the first time at which infection with the corresponding genotype was detected with a valid lineage assignment). During quality control, 37% of infections were excluded due to sequencing failure, insufficient coverage or poor-quality reads across the HPV genome, or a within-type lineage coinfection that could not be resolved or had an ambiguous position in the phylogenetic tree. Before the main analyses, we conducted analyses to determine whether selection bias was introduced, including comparisons of: the proportion of samples with sequencing results by HPV genotype across vaccination status (Supplementary Table 1); woman-level characteristics by sequencing result status (Supplementary Table 2); and the distribution of HPV positivity by vaccination status across sequencing result status (Supplementary Table 3). These metrics confirmed that our analytical samples were not substantially different from excluded samples, thus, our results are assumed to be generalizable to the full study population.

### Analytical cohort

We included women with at least one HPV DNA test result after two years post-enrollment who did not have an HPV type-specific outcome of interest during the initial two-year period to minimize misclassification of infections present at the time of recruitment that were not detected by our HPV genotyping assay. The two-year period was used because most (80%-90%) HPV infections spontaneously clear within two years^33^. The analytical cohort for the randomized trial phase (years 1–4) included women who received three doses of Cervarix (HPV-vaccine arm), and three doses of Havrix (HAV-vaccine arm); women who were free of type-specific infections in the two years following recruitment were genotyped at years 3 and 4. The analytical cohort for the observational LTFU phase (years 4-11) included women who received three doses of Cervarix during the randomized trial phase (HPV-vaccine arm) and women UCG; women who were free of type-specific infections during two years following their enrollment into the long-term follow-up phase (i.e., event-free during years 4-7) were genotyped at years 9 and 11.

### HPV DNA detection and genotyping

During the trial phase, DNA was extracted from cervical samples and tested for HPV DNA detection and genotyped using the SPF10 polymerase chain reaction primer system and DNA enzyme immunoassay detection of amplimers (DEIA) system (DDL Diagnostic Laboratory, Delft, Netherlands). Specifically, primers were used to amplify a fragment from the L1 region of HPV genotypes, after which the DEIA system detected the amplified products. All DEIA-positive SPF10 amplimers were used to identify HPV genotype by reverse hybridization with the LiPA25 HPV line probe assay (Labo Bio-medical Products, Rijswijk, Netherlands). During the long-term follow-up, HPV DNA detection was replaced with TypeSeq (National Cancer Institute Cancer Genomics Research Laboratory, Frederick, MD, USA) and genotyping was done using Ion S5 next-generation sequencing, followed by a custom Torrent Suite plugin analysis (Thermo Fisher Scientific, Waltham, MA, USA). A prior comparison of LiPA25 and TypeSeq found that the percent agreement between both genotyping methods was high for the four HPV types (HPV 31/33/35/45) in our study (total agreement range = 99.5%-99.8%; positive agreement range = 88.2%–93.3%)^34^. More detailed HPV DNA detection and genotyping methods are presented elsewhere^31,32^.

### HPV viral whole-genome sequencing

The first positive cervical sample of HPV31, 33, 35, and 45 for each woman was HPV whole-genome sequenced using custom Thermo Fisher Ion Torrent AmpliSeq HPV type-specific panels to amplify the entire genome, as previously described^35^. Briefly, custom overlapping degenerate primers were created to cover the entire viral genome of HPV31, 33, 35, or 45. After amplification, an Ion Torrent adapter-ligated library was generated following the manufacturer’s Ion AmpliSeq Library Preparation kit 2.0-96LV protocol with slight modifications (Life Technologies, Part #4480441). Raw sequencing reads were quality and adaptor trimmed and aligned to the HPV type-specific reference sequence from PAVE^36^ using the Torrent Mapping Alignment Program v5.0.13^37^. Individual nucleotide variants of each HPV type were called using the Torrent Variant Caller v.5.0.3 and annotated using snpEff v.3.6 c^38^.

### Classification of HPV variants

A consensus whole-genome sequence was created for each sample and HPV type and combined with type-specific lineage reference sequences from GenBank^39^. Phylogenetic trees were constructed using RAxML MPI v7.2.8.27^40^ and MEGAX^41^ with 1000 bootstraps. Lineages were assigned based on the tree topology and sample proximity to the reference lineage genomes and confirmed with individual SNP patterns. For each HPV type, lineages were categorized as: HPV31-lineage-A, B, or C; HPV33-lineage-A or B; HPV35-sublineage-A1 or A2; and HPV45-lineage-A or B. Notably, HPV35 consists predominately of one main lineage-A that is divided into two sublineages, A1 and A2^7^, but there is also a B lineage found in Asia^11^, but is not observed in our study population. For each HPV type, individual SNPs or variable positions within the L1 region were evaluated if they were observed in at least two samples. For each HPV type, the L1 region boundaries are presented in Supplementary Table 4. For L1-SNPs, we compared allele frequencies at each nucleotide position between HPV-vaccinated women and HPV-unvaccinated women using the most common nucleotide at each position as the referent group (Supplementary Table 5).

### Statistical analysis

Balanced baseline characteristics of women in the HPV-vaccine arm and HAV-vaccine arm have been demonstrated^31^. When the UCG was enrolled (the fourth year study visit of CVT), characteristics of the original control group were compared with UCG^32^. In the present study, among women who met our inclusion criteria, we described characteristics at the year four study visit (month 48 visit for the HPV-vaccine arm and HAV-vaccine arm and the baseline visit for UCG) by all vaccination status groups, including the HPV-vaccine arm, combined HPV-unvaccinated group, HAV-vaccine arm alone, and UCG alone.

For each HPV type irrespective of variant and stratified by variant, we reported the cumulative incidence over 11 years of follow-up, VE [*100 x (1-relative risk)*], and asymptotic 95% confidence intervals (95%CIs). We calculated the proportions of women in the randomized trial phase who were not infected through year 2 but tested positive for a specific genotype later, namely,

1 $$pTria{l}_{HPV-vaccinearm}=\frac{(Number\,of\,Cervarix-vaccinated\,women\,who\,tested\,positive\,for\,genotype\,in\,years\,3\,or\,4)}{(Number\,of\,Cervarix-vaccinated\,women\,who\,were\,negative\,for\,that\,genotype\,through\,year\,2)},$$

and

2 $$pTria{l}_{HAV-vaccinearm}=\frac{(Number\,of\,Havrix-vaccinated\,women\,who\,tested\,positive\,for\,genotype\,in\,years\,3\,or\,4)}{(Number\,of\,Havrix-vaccinated\,women\,who\,were\,negative\,for\,that\,genotype\,through\,year\,2)}.$$

For the LTFU phase, we calculated

3 $$pLTF{U}_{HPV-vaccinearm}=\frac{(Number\,of\,Cervarix-vaccinated\,women\,who\,tested\,positive\,for\,genotype\,in\,years\,9\,or\,11)}{(Number\,of\,Cervarix-vaccinated\,women\,who\,were\,negative\,for\,that\,genotype\,in\,years\,4-7)},$$

and

4 $$pLTF{U}_{UCG}=\frac{(Number\,of\,UCG\,women\,who\,tested\,positive\,for\,genotype\,in\,years\,9\,or\,11)}{(Number\,of\,UCG\,women\,who\,were\,negative\,for\,that\,genotype\,2\,years\,after\,recruitment)}.$$

We calculated relative risks for the trial phase, *pTrial*_*HPV-vaccine arm*_ */ pTrial*_*HAV-vaccine arm*_, and for the LTFU phase, *pLTFU*_*HPV-vaccine arm*_ */ pLTFU*_*UCG*_. To get a combined probability of infection for the control groups over the full 11-year follow-up period, we calculated *p*_*control*_ *= pTrial*_*HAV-vaccine arm*_ + *(1-pTrial*_*HAV-vaccine arm*_*) x pLTFU*_*UCG*_. Likewise, *p*_*HPV-vaccine arm*_ *= pTrial*_*HAV-vaccine arm*_ + *(1-pTrial*_*HAV-vaccine arm*_*) x pLTFU*_*HAV-vaccine arm*_. The combined relative risk was calculated by *p*_*HPV-vaccine arm*_ */ p*_*control*_. We resampled women with replacement to obtain bootstrap distributions of vaccine efficacy and vaccine efficacy ratio estimates^42^.

We identified ten individual L1-SNPs that were significantly associated with vaccination status (nine for HPV31 and one for HPV35) (Supplementary Table 5), for which VE was calculated stratified by nucleotide (e.g., nucleotide-T and nucleotide-C at SNP position 6862). To evaluate whether VE differed across variants, we calculated VE ratios between groups (e.g., HPV31-lineage-B compared to HPV31-lineage-A, or nucleotide-T compared to nucleotide-C at SNP position 6862). Statistical significance was determined if the VE 95%CI did not include zero, and VE ratio 95%CI did not include 1.0. We translated and annotated each DNA change (SNP) observed in L1 and mapped them to L1 loops to determine whether the SNP was located within a neutralization domain. Three-dimensional homology models for the only non-synonymous L1-SNP position 6372 (amino acid position 274) were created using the SWISS-MODEL (https://swissmodel.expasy.org/) to visualize potential structural changes related to this amino acid change.

Recognizing that the analysis includes different control groups over two study periods, we conducted a sensitivity analysis to compare VE between study phases to justify combining periods for a cumulative VE across the full 11 years. None of the VEs were significantly different between time points (all variants p-heterogeneity > 0.05, Supplementary Table 6) and follow-up times were similar during each study phase (Supplementary Table 7).

### Ethics and inclusion

Local researchers from Costa Rica were involved throughout the entire research process, including study design, study implementation, data ownership, data analysis, and manuscript preparation. The research is locally relevant to women in Costa Rica. Authorship roles and responsibilities were agreed upon prior to the conduct of this analysis. The project and resulting manuscript were reviewed by the Costa Rica HPV Vaccine Trial group.

### Reporting summary

Further information on research design is available in the Nature Research Reporting Summary linked to this article.

### Supplementary information

Supplementary Information

Reporting Summary

## Data Availability

Participant data can be shared with outside collaborators for research to understand more about the performance of the HPV vaccine, immune response to the vaccine, and broader study factors associated with the natural history of HPV infection and risk factors for infection and disease. Outside collaborators can apply to access our protocols and data from the blinded phase of the Costa Rica Vaccine Trial (NCT00128661). All of the L1 nucleotide sequences used in our study have been deposited in GenBank (accession numbers PP791977 - PP792123, PP792124 - PP792208, PP792209 - PP792340, and PP792341 - PP792520). A trial summary, current publications, and contact information are available online at: https://dceg.cancer.gov/research/who-we-study/cohorts/costa-rica-vaccine-trial.
