# Supplementary material for: Differential long-term bivalent HPV vaccine cross-protection by variants in the Costa Rica HPV vaccine trial
Source: NPJ Vaccines. 2024 Jun 8;9:101. doi: 10.1038/s41541-024-00896-y (PMC11162434; doi:10.1038/s41541-024-00896-y)
Supplement: Supplementary file 1 — Supplementary Information [file 41541_2024_896_MOESM1_ESM.pdf]

Supplementary Table 1. Proportion of samples with sequencing results by HPV genotype and vaccination status.

	Overall		HPV-Vaccine Arm		Combined HPV-Unvaccinated Group		HAV-Vaccine Arm		UCG	
**Characteristic**	**Total samples tested (visit-level) n (%)**	**Samples with results n (%)**	**Total samples tested (visit-level) n (%)**	**Samples with results n (%)**	**Total samples tested (visit-level) n (%)**	**Samples with results n (%)**	**Total samples tested (visit-level) n (%)**	**Samples with results n (%)**	**Total samples tested (visit-level) n (%)**	**Samples with results n (%)**
Total	860	544 (63.3)	246	141 (57.3)	614	403 (65.6)	321	209 (65.1)	293	194 (66.2)
Genotype										
HPV 31	305 (35.5)	180 (59.0)	69 (28.0)	28 (40.6)	236 (38.4)	152 (64.4)	136 (42.4)	88 (64.7)	100 (34.1)	64 (64.0)
HPV 33	132 (15.3)	85 (64.4)	54 (22.0)	35 (64.8)	78 (12.7)	50 (64.1)	47 (14.6)	27 (57.4)	31 (10.6)	23 (74.2)
HPV 35	215 (25.0)	147 (68.4)	89 (36.2)	61 (68.5)	126 (20.5)	86 (68.3)	59 (18.4)	39 (66.1)	67 (22.9)	47 (70.1)
HPV 45	208 (24.2)	132 (63.5)	34 (13.8)	17 (50.0)	174 (28.3)	115 (66.1)	79 (24.6)	55 (69.6)	95 (32.4)	60 (63.2)

HAV = hepatitis A virus, HPV = human papillomavirus, UCG = screening-only, observational unvaccinated control group.

Results from this table show that the proportion of sample results for each genotype do not qualitatively differ between vaccination groups.

Supplementary Table 2. Characteristics of women at year 4 (visit 48), by sequencing result status.

Characteristic	Inconclusive Results for All Tests	Had Results for All Tests	Some Had Results But Not All
Total, N	243	458	56
HPV vaccination status, n (%)			
HPV-vaccine arm	83 (34.2)	119 (26.0)	17 (30.4)
Combined HPV-unvaccinated group	160 (65.8)	339 (74.0)	39 (69.6)
HAV-vaccine arm	79 (32.5)	174 (38.0)	24 (42.9)
UCG	81 (33.3)	165 (36.0)	15 (26.8)
Age, years, n (%)			
<26	118 (48.6)	241 (52.6)	23 (41.1)
26+	121 (49.8)	211 (46.1)	30 (53.6)
No visit	4 (1.6)	6 (1.3)	3 (5.4)
Median, IQR (range)	26, 24-28 (22-32)	25, 24-27 (21-32)	26, 24-28 (22-30)
HPV positivity, n (% [a]; 95% CI)			
HPV 16/18	24 (9.9; 6.6-14.1)	33 (7.2; 5.1-9.9)	7 (12.5; 5.6-23.2)
Other oncogenic HPV	103 (42.4; 36.3-48.7)	221 (48.3; 43.7-52.8)	34 (60.7; 47.5-72.8)
Non oncogenic HPV	80 (32.9; 27.2-39.0)	147 (32.1; 27.9-36.5)	19 (33.9; 22.5-47.0)
No HPV	83 (34.2; 28.4-40.3)	157 (34.3; 30.0-38.7)	10 (17.9; 9.4-29.5)
No result	4 (1.6; 0.5-3.9)	6 (1.3; 0.5-2.7)	3 (5.4; 1.4-13.9)
Number of cross-protected type 31/33/35/45 infections for which the woman joined the analysis, n (%)			
1	228 (93.8)	433 (94.5)	N/A
2	15 (6.2)	24 (5.2)	50 (89.3)
3	0 (0.0)	1 (0.2)	6 (10.7)

HAV = hepatitis A virus, HPV = human papillomavirus, IQR = interquartile range, N/A = not applicable; UCG = screening-only, observational unvaccinated control group.
[a] The sum of the percentages for HPV positivity does not equal 100 because women who had multiple HPV type infections were counted in multiple rows.

Results from this table suggest that characteristics of women do not qualitatively differ by sequencing result status.

Supplementary Table 3. HPV positivity by vaccination status and sequencing result status.

HPV positivity n (% [a]; 95% CI)	No Results for All Tests				Had Results for All Tests				Some Had Results But Not All			
	HPV-Vaccine Arm	Combined HPV-Unvaccinated Group	HAV-Vaccine Arm	UCG	HPV-Vaccine Arm	Combined HPV-Unvaccinated Group	HAV-Vaccine Arm	UCG	HPV-Vaccine Arm	Combined HPV-Unvaccinated Group	HAV-Vaccine Arm	UCG
Total, N	83	160	79	81	119	339	174	165	17	39	24	15
HPV 16/18	0 (0.0; 0.0-3.5)	24 (15.0; 10.1-21.2)	13 (16.5; 9.5-25.9)	11 (13.6; 7.4-22.4)	2 (1.7; 0.3-5.4)	31 (9.1; 6.4-12.6)	18 (10.3; 6.4-15.6)	13 (7.9; 4.5-12.8)	1 (5.9; 0.3-25.8)	6 (15.4; 6.5-29.3)	6 (25.0; 10.8-44.9)	0 (0.0; 0.0-18.1)
Other oncogenic HPV	45 (54.2; 43.4-64.7)	58 (36.3; 29.1-43.9)	40 (50.6; 39.7-61.5)	18 (22.2; 14.2-32.2)	56 (47.1; 38.2-56.0)	165 (48.7; 43.4-54.0)	125 (71.8; 64.8-78.1)	40 (24.2; 18.2-31.2)	11 (64.7; 40.5-84.3)	23 (59.0; 43.2-73.5)	20 (83.3; 64.5-94.5)	3 (20.0; 5.4-45.3)
Non-oncogenic HPV	32 (38.6; 28.6-49.3)	48 (30.0; 23.3-37.4)	30 (38.0; 27.8-49.0)	18 (22.2; 14.2-32.2)	46 (38.7; 30.2-47.6)	101 (29.8; 25.1-34.8)	61 (35.1; 28.2-42.4)	40 (24.2; 18.2-31.2)	5 (29.4; 11.7-53.7)	14 (35.9; 22.1-51.7)	9 (37.5; 20.1-57.8)	5 (33.3; 13.4-59.2)
No HPV	22 (26.5; 17.9-36.8)	61 (38.1; 30.8-45.8)	20 (25.3; 16.7-35.8)	41 (50.6; 39.8-61.4)	44 (37.0; 28.7-45.9)	113 (33.3; 28.5-38.5)	18 (10.3; 6.4-15.6)	95 (57.6; 49.9-65.0)	2 (11.8; 2.0-33.7)	8 (20.5; 10.0-35.3)	1 (4.2; 0.2-18.9)	7 (46.7; 23.2-71.3)
No result	2 (2.4; 0.4-7.7)	2 (1.3; 0.2-4.1)	2 (2.5; 0.4-8.1)	0 (0.0; 0.0-3.6)	0 (0.0; 0.0-2.5)	6 (1.8; 0.7-3.6)	6 (3.4; 1.4-7.0)	0 (0.0; 0.0-1.8)	1 (5.9; 0.3-25.8)	2 (5.1; 0.9-15.9)	2 (8.3; 1.4-24.9)	0 (0.0; 0.0-18.1)

CI = confidence interval, HAV = hepatitis A virus, HPV = human papillomavirus, IQR = interquartile range, UCG = screening-only, observational unvaccinated control group.

[a] The sum of the percentages for HPV positivity does not equal 100 because women who had multiple type infections were counted in multiple rows.

Results from this table suggest that HPV positivity by vaccination status and sequencing result status do not qualitatively differ. Given the exploratory nature of this investigation into possible selection bias by testing status, we did not adjust for multiple comparisons.

Supplementary Table 4. L1 boundary positions and proportion of polymorphisms by HPV type.

HPV Type	L1 Lower Bound	L1 Upper Bound	Nucleotides with Variability [a] n (%)
HPV 31	5552	7066	42 (2.8)
HPV 33	5594	7093	25 (1.7)
HPV 35	5601	7109	26 (1.7)
HPV 45	5608	7149	35 (2.3)

HPV = human papillomavirus.

[a] Polymorphism observed in at least two samples.

Supplementary Table 5. Significant SNPs in the L1 gene region of HPV 31/33/35/45 [a] that had variability in at least two samples.

Position	Minor Allele	Minor Allele Frequency	Number of Observations	Nucleotide A	Nucleotide T	Nucleotide C	Nucleotide G	Odds Ratio [b] (95% CI)	P-value
HPV 31									
5921	T	0.24	179	0	43	136	0	0.21 (0.05-0.91)	0.04
6127	G	0.22	179	139	0	0	40	2.72 (1.15-6.43)	0.02
6238	A	0.22	177	39	138	0	0	2.80 (1.18-6.63)	0.02
6367	C	0.23	177	0	136	41	0	0.22 (0.05-0.96)	0.04
6372	C	0.24	177	135	0	42	0	0.21 (0.05-0.92)	0.04
6379	G	0.16	179	150	0	0	29	3.10 (1.23-7.80)	0.02
6772	A	0.21	180	38	0	0	142	3.00 (1.26-7.11)	0.01
6796	G	0.24	180	137	0	0	43	0.21 (0.05-0.92)	0.04
6862	T	0.16	180	0	28	152	0	3.32 (1.31-8.38)	0.01
HPV 35									
5939	G	0.23	145	112	0	0	33	0.37 (0.15-0.89)	0.03

CI = confidence interval; HPV = human papillomavirus; SNP = single nucleotide polymorphism.

[a] SNPs in the L1 gene region were assessed for HPV 31, 33, 35, and 45, but significant SNPs were only identified for HPV 31 and 35.

[b] The odds ratios compare allele frequencies at each nucleotide position between HPV-vaccinated and HPV-unvaccinated women using the most common nucleotide at each position as the referent group; thus, the interpretation is the odds of having the minor allele at the specific nucleotide position in HPV-vaccinated women compared to the odds among HPV-unvaccinated women.

Supplementary Table 6. Comparison of vaccine efficacy during the randomized trial phase and the observational long-term follow-up phase, by variant.

	Randomized Trial Phase					Observational Long-Term Follow-Up Phase					
	HPV-Vaccine Arm		HAV-Vaccine Arm			HPV-Vaccine Arm		UCG			
	n	Rate (95% CI)	n	Rate (95% CI)	VE (95% CI)	n	Rate (95% CI)	n	Rate (95% CI)	VE (95% CI)	p-value [a]
HPV 31 (Total N)	2665		2637			2163		2382			
Overall	13	4.9 (2.7, 8.1)	88	33.4 (27.0, 40.8)	85.4 (74.5, 92.1)	15	6.9 (4.0, 11.2)	64	26.9 (20.9, 34.0)	74.2 (55.6, 85.7)	0.21
HPV 31 Lineage A	0	0.0 (0.0, 1.1)	26	9.9 (6.6, 14.2)	100.0 (87.9, 100.0)	2	0.9 (0.2, 3.1)	15	6.3 (3.7, 10.1)	85.3 (43.9, 97.7)	0.30
HPV 31 Lineage B	7	2.6 (1.1, 5.2)	19	7.2 (4.5, 11.0)	63.5 (15.2, 85.7)	4	1.8 (0.6, 4.5)	10	4.2 (2.1, 7.5)	56.0 (-37.1, 88.0)	0.84
HPV 31 Lineage C	6	2.3 (0.9, 4.7)	43	16.3 (12.0, 21.7)	86.2 (69.2, 94.7)	9	4.2 (2.0, 7.6)	39	16.4 (11.8, 22.1)	74.6 (49.1, 88.4)	0.33
5921 = T	0	0.0 (0.0, 1.1)	26	9.9 (6.6, 14.2)	100.0 (87.9, 100.0)	2	0.9 (0.2, 3.1)	15	6.3 (3.7, 10.1)	85.3 (43.9, 97.7)	0.30
5921 = C	13	4.9 (2.7, 8.1)	61	23.1 (17.9, 29.4)	78.9 (62.5, 88.8)	13	6.0 (3.3, 10.0)	49	20.6 (15.4, 26.9)	70.8 (47.2, 84.7)	0.49
6127 = A	6	2.3 (0.9, 4.7)	68	25.8 (20.2, 32.4)	91.3 (81.0, 96.6)	11	5.1 (2.7, 8.8)	53	22.3 (16.9, 28.8)	77.1 (57.4, 88.6)	0.11
6127 = G	7	2.6 (1.1, 5.2)	20	7.6 (4.8, 11.5)	65.4 (20.1, 86.4)	4	1.8 (0.6, 4.5)	10	4.2 (2.1, 7.5)	56.0 (-37.1, 88.0)	0.79
6238 = T	6	2.3 (0.9, 4.7)	68	25.8 (20.2, 32.4)	91.3 (81.0, 96.6)	11	5.1 (2.7, 8.8)	53	22.3 (16.9, 28.8)	77.1 (57.4, 88.6)	0.11
6238 = A	7	2.6 (1.1, 5.2)	18	6.8 (4.2, 10.6)	61.5 (9.8, 85.0)	4	1.8 (0.6, 4.5)	10	4.2 (2.1, 7.5)	56.0 (-37.1, 88.0)	0.88
6367 = C	0	0.0 (0.0, 1.1)	26	9.9 (6.6, 14.2)	100.0 (87.9, 100.0)	2	0.9 (0.2, 3.1)	13	5.5 (3.0, 9.1)	83.1 (33.9, 97.4)	0.30
6367 = T	13	4.9 (2.7, 8.1)	60	22.8 (17.6, 29.0)	78.6 (61.8, 88.7)	13	6.0 (3.3, 10.0)	50	21.0 (15.8, 27.4)	71.4 (48.3, 85.0)	0.53
6372 = C	0	0.0 (0.0, 1.1)	26	9.9 (6.6, 14.2)	100.0 (87.9, 100.0)	2	0.9 (0.2, 3.1)	14	5.9 (3.3, 9.6)	84.3 (39.3, 97.6)	0.30
6372 = A	13	4.9 (2.7, 8.1)	60	22.8 (17.6, 29.0)	78.6 (61.8, 88.7)	13	6.0 (3.3, 10.0)	49	20.6 (15.4, 26.9)	70.8 (47.2, 84.7)	0.51
6379 = A	7	2.6 (1.1, 5.2)	73	27.7 (21.9, 34.5)	90.5 (80.4, 96.0)	12	5.5 (3.0, 9.4)	57	23.9 (18.3, 30.7)	76.8 (57.8, 88.1)	0.11
6379 = G	6	2.3 (0.9, 4.7)	14	5.3 (3.0, 8.7)	57.6 (-8.4, 85.0)	3	1.4 (0.4, 3.8)	7	2.9 (1.3, 5.8)	52.8 (-79.6, 90.1)	0.92
6772 = G	6	2.3 (0.9, 4.7)	70	26.5 (20.9, 33.2)	91.5 (81.6, 96.7)	11	5.1 (2.7, 8.8)	54	22.7 (17.2, 29.3)	77.6 (58.2, 88.8)	0.11
6772 = A	7	2.6 (1.1, 5.2)	18	6.8 (4.2, 10.6)	61.5 (9.8, 85.0)	4	1.8 (0.6, 4.5)	10	4.2 (2.1, 7.5)	56.0 (-37.1, 88.0)	0.88
6796 = G	0	0.0 (0.0, 1.1)	26	9.9 (6.6, 14.2)	100.0 (87.9, 100.0)	2	0.9 (0.2, 3.1)	15	6.3 (3.7, 10.1)	85.3 (43.9, 97.7)	0.30
6796 = A	13	4.9 (2.7, 8.1)	62	23.5 (18.2, 29.8)	79.3 (63.1, 89.0)	13	6.0 (3.3, 10.0)	49	20.6 (15.4, 26.9)	70.8 (47.2, 84.7)	0.47
6862 = C	7	2.6 (1.1, 5.2)	74	28.1 (22.3, 34.9)	90.6 (80.7, 96.0)	12	5.5 (3.0, 9.4)	58	24.3 (18.7, 31.1)	77.2 (58.6, 88.3)	0.12
6862 = T	6	2.3 (0.9, 4.7)	14	5.3 (3.0, 8.7)	57.6 (-8.4, 85.0)	3	1.4 (0.4, 3.8)	6	2.5 (1.0, 5.2)	44.9 (-121.2, 88.8)	0.83
HPV 33 (Total N)	2775		2810			2192		2500			
Overall	13	4.7 (2.6, 7.8)	27	9.6 (6.5, 13.8)	51.2 (6.4, 75.6)	22	10.0 (6.5, 14.9)	23	9.2 (6.0, 13.6)	-9.1 (-97.0, 39.7)	0.12
HPV 33 Lineage A	11	4.0 (2.1, 6.9)	24	8.5 (5.6, 12.5)	53.6 (6.3, 78.1)	22	10.0 (6.5, 14.9)	20	8.0 (5.0, 12.1)	-25.5 (-132.3, 31.9)	0.08
HPV 33 Lineage B	2	0.7 (0.1, 2.4)	3	1.1 (0.3, 2.9)	32.5 (-354.0, 92.0)	0	0.0 (0.0, 1.4)	3	1.2 (0.3, 3.3)	100.0 (-95.5, 100.0)	0.59
HPV 35 (Total N)	2757		2779			2182		2489			
Overall	31	11.2 (7.8, 15.7)	39	14.0 (10.1, 18.9)	19.9 (-28.5, 50.4)	30	13.7 (9.5, 19.3)	47	18.9 (14.1, 24.8)	27.2 (-14.8, 54.4)	0.78
HPV 35 Lineage A1	30	10.9 (7.5, 15.3)	38	13.7 (9.8, 18.5)	20.4 (-28.5, 51.1)	27	12.4 (8.3, 17.7)	44	17.7 (13.0, 23.4)	30.0 (-12.7, 57.1)	0.72
HPV 35 Lineage A2	1	0.4 (0.0, 1.8)	1	0.4 (0.0, 1.8)	-0.8 (-3831.1, 97.4)	3	1.4 (0.3, 3.7)	3	1.2 (0.3, 3.3)	-14.1 (-563.9, 80.4)	0.99
5939 = G	3	1.1 (0.3, 3.0)	9	3.2 (1.6, 5.9)	66.4 (-18.9, 92.7)	5	2.3 (0.8, 5.1)	16	6.4 (3.8, 10.2)	64.4 (6.1, 88.3)	0.95
5939 = A	28	10.2 (6.9, 14.4)	30	10.8 (7.4, 15.2)	5.9 (-58.1, 44.1)	24	11.0 (7.2, 16.1)	30	12.1 (8.3, 16.9)	8.7 (-56.4, 47.2)	0.94
HPV 45 (Total N)	2747		2746			2194		2461			
Overall	11	4.0 (2.1, 6.9)	55	20.0 (15.3, 25.8)	80.0 (62.8, 90.0)	6	2.7 (1.1, 5.7)	60	24.4 (18.8, 31.1)	88.8 (75.5, 95.6)	0.31
HPV 45 Lineage A	6	2.2 (0.9, 4.5)	27	9.8 (6.6, 14.1)	77.8 (48.4, 91.6)	4	1.8 (0.6, 4.4)	32	13.0 (9.1, 18.1)	86.0 (63.3, 95.8)	0.55
HPV 45 Lineage B	5	1.8 (0.7, 4.0)	28	10.2 (6.9, 14.5)	82.1 (56.3, 93.9)	2	0.9 (0.2, 3.0)	28	11.4 (7.7, 16.2)	92.0 (71.4, 98.7)	0.41

CI = confidence interval; HAV = hepatitis A virus; HPV = human papillomavirus; UCG = screening-only, observational unvaccinated control group; VE = vaccine efficacy.

[a] The p-value represents a test for heterogeneity between the vaccine efficacy in the randomized trial phase compared to the vaccine efficacy in the observational long-term follow-up phase.

Supplementary Table 7. Comparison of follow-up time and testing between women included in the analysis from the randomized control trial phase and the long-term follow-up phase.

	Randomized Control Trial Phase		Long-Term Follow-Up Phase	
	**HPV-Vaccine Arm**	**HAV-Vaccine Arm**	**HPV-Vaccine Arm**	**UCG**
Total women (N)	2846	2909	2222 [a]	2556
Months of follow up				
Median (IQR)	20.5 (17.6-23.5)	20.5 (17.8-23.5)	52.7 (46.8-60.4)	49.6 (42.4-56.1)
Mean (SD)	20.97 (6.13)	21.22 (6.28)	52.09 (13.44)	49.09 (13.29)
Number of PCR tests				
Median (IQR)	2 (2-2)	2 (2-3)	2 (2-3)	2 (2-3)
Mean (SD)	2.28 (0.82)	2.33 (0.85)	2.63 (1.68)	2.93 (1.97)

HAV = hepatitis A virus; HPV = human papillomavirus; IQR = interquartile range; PCR = polymerase chain reaction; SD = standard deviation; UCG = screening-only, observational unvaccinated control group.

[a] The HPV arm in the long-term follow-up phase is smaller than the HPV arm in the randomized control trial phase due to women who were either lost to follow-up or not invited to participate in the long-term follow-up phase.
