# Supplementary material for: Differential long-term bivalent HPV vaccine cross-protection by variants in the Costa Rica HPV vaccine trial
Source: NPJ Vaccines. 2024 Jun 8;9:101. doi: 10.1038/s41541-024-00896-y (PMC11162434; doi:10.1038/s41541-024-00896-y)
Supplement: Supplementary file 2 — Reporting Summary [file 41541_2024_896_MOESM2_ESM.pdf]

nature portfolio

Corresponding author(s): Jaimie Z. Shing; Division of Cancer Epidemiology and Genetics, National Cancer Institute; 9609 Medical Center Drive, Rockville, MD 20892; E-mail: jaimie.shing@nih.gov; Phone: 240-276-7183

Last updated by author(s): May 14, 2024

Reporting Summary

Nature Portfolio wishes to improve the reproducibility of the work that we publish. This form provides structure for consistency and transparency in reporting. For further information on Nature Portfolio policies, see our Editorial Policies and the Editorial Policy Checklist.

Statistics

For all statistical analyses, confirm that the following items are present in the figure legend, table legend, main text, or Methods section.

n/a	Confirmed	
☐	☒	The exact sample size (*n*) for each experimental group/condition, given as a discrete number and unit of measurement
☐	☒	A statement on whether measurements were taken from distinct samples or whether the same sample was measured repeatedly
☐	☒	The statistical test(s) used AND whether they are one- or two-sided<br>*Only common tests should be described solely by name; describe more complex techniques in the Methods section.*
☐	☒	A description of all covariates tested
☐	☒	A description of any assumptions or corrections, such as tests of normality and adjustment for multiple comparisons
☐	☒	A full description of the statistical parameters including central tendency (e.g. means) or other basic estimates (e.g. regression coefficient) AND variation (e.g. standard deviation) or associated estimates of uncertainty (e.g. confidence intervals)
☐	☒	For null hypothesis testing, the test statistic (e.g. *F*, *t*, *r*) with confidence intervals, effect sizes, degrees of freedom and *P* value noted<br>*Give P values as exact values whenever suitable.*
☒	☐	For Bayesian analysis, information on the choice of priors and Markov chain Monte Carlo settings
☐	☒	For hierarchical and complex designs, identification of the appropriate level for tests and full reporting of outcomes
☐	☒	Estimates of effect sizes (e.g. Cohen's *d*, Pearson's *r*), indicating how they were calculated

Our web collection on statistics for biologists contains articles on many of the points above.

Software and code

Policy information about availability of computer code

Data collection	no software was used
Data analysis	no software was used

For manuscripts utilizing custom algorithms or software that are central to the research but not yet described in published literature, software must be made available to editors and reviewers. We strongly encourage code deposition in a community repository (e.g. GitHub). See the Nature Portfolio guidelines for submitting code & software for further information.

Data

Policy information about availability of data

All manuscripts must include a data availability statement. This statement should provide the following information, where applicable:

- Accession codes, unique identifiers, or web links for publicly available datasets
- A description of any restrictions on data availability
- For clinical datasets or third party data, please ensure that the statement adheres to our policy

Participant data can be shared with outside collaborators for research to understand more about the performance of the HPV vaccine, immune response to the vaccine, and broader study factors associated with the natural history of HPV infection and risk factors for infection and disease. Outside collaborators can apply to access our protocols and data from the blinded phase of the Costa Rica Vaccine Trial (NCT00128661). All of the L1 nucleotide sequences used in our study have

been deposited in GenBank (accession numbers PP791977 - PP792123, PP792124 - PP792208, PP792209 - PP792340, and PP792341 - PP792520). A trial summary, current publications, and contact information are available online at: https://dceg.cancer.gov/research/who-we-study/cohorts/costa-rica-vaccine-trial.

Research involving human participants, their data, or biological material

Policy information about studies with human participants or human data. See also policy information about sex, gender (identity/presentation), and sexual orientation and race, ethnicity and racism.

Reporting on sex and gender	The Costa Rica HPV Vaccine trial is among women only because it was designed to evaluate the efficacy of Cervarix against HPV infections and HPV-associated cervical neoplasia.
Reporting on race, ethnicity, or other socially relevant groupings	We do not report race or ethnicity in the study because the majority (84%) of the Costa Rican population is White or Mestizo.
Population characteristics	The analytical cohort included 2846 HPV-vaccinated and 5465 HPV-unvaccinated women (2909 from the Hepatitis A vaccine arm and 2556 from the unvaccinated control group). At the fourth-year study visit, both groups had the same median age (26 years, interquartile range=24-28 years).
Recruitment	During 2004-2005, before Cervarix licensure, 7466 Costa Rican women aged 18-25 years were enrolled and randomized (1:1) to receive three doses of either Cervarix or the control hepatitis A virus (HAV) Havrix® vaccine in the Costa Rica HPV Vaccine Trial. The recruitment methods for this have been thoroughly described elsewhere (doi:10.1016/j.vaccine.2008.07.002).
Ethics oversight	Research activity for the Costa Rica HPV Vaccine Trial was approved by Institutional Review Boards of Instituto Costarricense de Investigación y Enseñanza en Nutrición y Salud in Costa Rica and the US National Cancer Institute (Bethesda, MD, USA).

Note that full information on the approval of the study protocol must also be provided in the manuscript.

Field-specific reporting

Please select the one below that is the best fit for your research. If you are not sure, read the appropriate sections before making your selection.

☒ Life sciences ☐ Behavioural & social sciences ☐ Ecological, evolutionary & environmental sciences

For a reference copy of the document with all sections, see nature.com/documents/nr-reporting-summary-flat.pdf

Life sciences study design

All studies must disclose on these points even when the disclosure is negative.

Sample size	Sample size and power calculations for the randomized, blinded phase of the trial are reported elsewhere (doi:10.1016/j.vaccine.2008.07.002). For the observational long-term follow-up phase, the target enrollment for unvaccinated participants was 3000 to provide a comparable sample size to the original hepatitis A vaccine control group in the Costa Rica HPV Vaccine Trial.
Data exclusions	During quality control, 37% of infections were excluded due to sequencing failure, insufficient coverage or poor-quality reads across the HPV genome, or a within-type lineage coinfection that could not be resolved or had an ambiguous position in the phylogenetic tree.
Replication	When the serum samples were obtained for this analysis, we randomly selected 10% for quality control.
Randomization	Participants were randomly assigned (1:1) to receive an HPV 16/18 AS04-adjuvanted vaccine or control hepatitis A vaccine, using a blocked randomization method, with permuted block sizes of 14, 16, and 18. The allocation sequence was generated using SAS (version 8.2) by staff at the National Cancer Institute. Allocation was concealed to participants, study personnel, and investigators, and was maintained throughout the 4-year randomised phase of the trial, after which participants were informed of their vaccination status and participants in the control group were offered the HPV vaccine. Detailed randomization and masking procedures have been described elsewhere (doi:10.1016/j.vaccine.2008.07.002). Participants in the long-term follow-up study were not masked because a screening only, observational unvaccinated group was enrolled by design.
Blinding	The original Costa Rica HPV Vaccine Trial was a randomized, double-blinded, community-based trial.

Reporting for specific materials, systems and methods

We require information from authors about some types of materials, experimental systems and methods used in many studies. Here, indicate whether each material, system or method listed is relevant to your study. If you are not sure if a list item applies to your research, read the appropriate section before selecting a response.

Materials & experimental systems

n/a	Involved in the study
☒	☐ Antibodies
☒	☐ Eukaryotic cell lines
☒	☐ Palaeontology and archaeology
☒	☐ Animals and other organisms
☐	☒ Clinical data
☒	☐ Dual use research of concern
☒	☐ Plants

Methods

n/a	Involved in the study
☒	☐ ChIP-seq
☒	☐ Flow cytometry
☒	☐ MRI-based neuroimaging

Clinical data

Policy information about clinical studies

All manuscripts should comply with the ICMJE guidelines for publication of clinical research and a completed CONSORT checklist must be included with all submissions.

Clinical trial registration

These studies are registered with ClinicalTrials.gov, NCT00128661 and NCT00867464

Study protocol

The protocols are in the supplements of many of the papers from the Costa Rica HPV Vaccine Trial group, such as doi.org/10.1016/S1470-2045(22)00291-1. We also included the protocol from the Costa Rica HPV Vaccine Trial and Long-Term Follow-up Study as a separate attachment.

Data collection

During 2004-2005, before Cervarix licensure, 7466 Costa Rican women aged 18-25 years were enrolled and randomized (1:1) to receive three doses of either Cervarix or the control hepatitis A virus (HAV) Havrix® vaccine in the Costa Rica HPV Vaccine Trial. The recruitment methods for this have been thoroughly described elsewhere (doi:10.1016/j.vaccine.2008.07.002). During the randomized trial phase, serum samples were collected at enrollment and at annual follow-up visit. For sexually experienced women, cervical samples were collected using a Cervex-Brush (Rovers Medical Devices BV, Oss, Netherlands) and rinsed in PreservCyt solution (Hologic, Marlborough, MA, USA) for cytology and HPV DNA testing. During the observational long-term follow-up phase, participants in both the HPV-vaccine arm and unvaccinated control group were seen biennially, and cervical samples were collected at each of these routine clinic visits. In both the trial phase and the LTFU phase, women with low-grade cervical abnormalities were seen every six months, while women with evidence of high-grade cervical abnormalities were referred to colposcopy for evaluation and treatment, as needed.

Outcomes

The primary outcomes were incident detection of HPV31, 33, 35, and/or 45 in cervical exfoliated cells. Each HPV-type was treated as an independent analysis; women who were infected with multiple cross-protected HPV types could contribute to each analysis. Lineage and SNPs were only determined at a single point (the first time at which infection with the corresponding genotype was detected with a valid lineage assignment). A consensus whole-genome sequence was created for each sample and HPV type and combined with type-specific lineage reference sequences from GenBank. Phylogenetic trees were constructed using RAxML MPI v7.2.8.27 and MEGAX with 1000 bootstraps. Lineages were assigned based on the tree topology and sample proximity to the reference lineage genomes and confirmed with individual SNP patterns.

Plants

Seed stocks

Report on the source of all seed stocks or other plant material used. If applicable, state the seed stock centre and catalogue number. If plant specimens were collected from the field, describe the collection location, date and sampling procedures.

Novel plant genotypes

Describe the methods by which all novel plant genotypes were produced. This includes those generated by transgenic approaches, gene editing, chemical/radiation-based mutagenesis and hybridization. For transgenic lines, describe the transformation method, the number of independent lines analyzed and the generation upon which experiments were performed. For gene-edited lines, describe the editor used, the endogenous sequence targeted for editing, the targeting guide RNA sequence (if applicable) and how the editor was applied.

Authentication

Describe any authentication procedures for each seed stock used or novel genotype generated. Describe any experiments used to assess the effect of a mutation and, where applicable, how potential secondary effects (e.g. second site T-DNA insertions, mosiacism, off-target gene editing) were examined.
